# Effects of 5-aminolevulinic acid photodynamic therapy for cervical low-grade squamous intraepithelial lesions with HR-HPV infections

**DOI:** 10.3389/fmed.2023.1301440

**Published:** 2024-02-09

**Authors:** Yu Li, Jing Chen, Yan Hu, Qingyang Xu, Rongzhen Jiang, Yincheng Teng, Yanli Xu, Li Ma

**Affiliations:** Department of Obstetrics and Gynecology, Shanghai Sixth People's Hospital Affiliated to Shanghai Jiao Tong University School of Medicine, Shanghai, China

**Keywords:** 5-aminolevulinic acid (5-ALA), photodynamic therapy (PDT), high-risk HPV (HR-HPV), low-grade squamous intraepithelial lesions (LSIL), cervical intraepithelial neoplasia (CIN)

## Abstract

**Objective:**

To determine the effectiveness and safety of 5-aminolevulinic acid mediated photodynamic therapy (5-ALA PDT) in HR-HPV infected patients with cervical low-grade squamous intraepithelial lesions (LSIL) and to explore possible factors affecting treatment outcomes.

**Methods:**

This retrospective study included 96 patients with histologically confirmed cervical LSIL and high-risk human papillomavirus (HR-HPV) infection. They received 5-ALA PDT treatment once a week for a total of 3 courses. All patients were evaluated by cytology tests, HPV DNA assay, colposcopy, and biopsy at 2 weeks, 3 months, and 6 months checkpoint. The chi-square test were used to evaluate the differences in various clinical data, and a *p* value <0.05 was considered statistically significant.

**Results:**

At 2 weeks, 3 months, and 6 months checkpoint, colposcopies showed that the cervical iodine-unstained area under VILI (visual inspection with Lugol’s iodine) significantly reduced (*p* < 0.01) with no structure changes. At 3 months and 6 months checkpoint, the pathological regression rate reached 87.5% (84/96) and 94.79% (91/96), while the HR-HPV clearance rates reached 80.21% (77/96) and 93.75% (90/96) respectively. We also examined the efficacy in the HPV 16/18-related group and non-HPV 16/18-related group. The HR-HPV clearance rate in the HPV16/18 group [94.87% (37/39)] was significantly higher than that of the non-HPV 16/18 group [70.17% (40/57)]. However, at 6 months after treatment, the clearance rate of the HPV 16/18 group [94.87% (37/39)] showed no statistical difference from the non-HPV 16/18 group [92.30% (53/57)].

**Conclusion:**

Topical 5-ALA PDT can effectively eliminate HR-HPV infection and treat low-grade cervical squamous intraepithelial lesions, it offers an alternative treatment option for patients with LSIL, especially for those with fertility requirements and who wish to preserve cervical structure or function.

## 1 Introduction

Cervical low-grade squamous intraepithelial lesions (LSIL) are low-risk cervical histopathological changes caused by HPV infection, including CIN 1 and condyloma lesions. At present, LISL treatment options contain observation and excision of lesions. LSIL has a high natural regression rate of about 60%, still 30% of the lesions would persist, and 10% would progress to HSIL (1, 2). Another meta-analysis showed that 48% of LSIL resolved spontaneously; 21% will progress to HSIL, and 0.15% of LSIL will progress to invasive cervical tumor (3). Till now, the therapeutic effect of antiviral drugs in CIN and HR-HPV infection is not thorough. LSIL patients experienced great anxiety and fear during follow-ups. In that way, they are more inclined to choose active elimination of HR-HPV infection and treatment of cervical lesions (4, 5).

Surgical excision therapies are lasers, a loop electrosurgical procedure, a cold knife conization, etc., which may lead to cervical structural changes and decreased function. Hence, these invasive treatments can cause obstetrical problems and are not suitable for patients with fertility requirements. Photodynamic therapy (PDT) is a combination of photosensitizer and photodynamic therapeutic apparatus to treat diseases. It works by using visible light to activate a photosensitizer to produce activated oxygen species that help destroy the lesions (6). The photosensitizer 5-aminolaevulinic acid (5-ALA) can be enriched in HPV-infected tissues and transformed into PpIX. After 635 nm red light irradiation, the photochemical reaction caused apoptosis and necrosis of the dysplasia cells, without affecting the physiological function of normal tissues. Due to PDT’s non-invasive and reproducible characteristics, it may offer an alternative for CIN and HPV infection (7). Many studies have been conducted on the clinical efficacy and safety of PDT in the treatment of cervical LSIL. The pathological complete response rate of patients after treatment ranges from 31 to 95%, and the HR-HPV clearance rate also ranges from 54 to 80% (8–10). This difference may be related to age, vaginal micro-environment, dosage of ALA, incubation time, exposure time of irradiation, treatment session, treatment intervals, and duration of follow-ups (11). Chinese expert consensus published in 2022 recommended PDT protocols for female lower genital tract diseases, but there are no authoritative international guidelines at present (12). Therefore, multi-center randomized controlled trials with large samples are still in demand.

Our study aims to evaluate the therapeutic effect of 5-ALA PDT on patients with cervical LSIL from the aspects of cervical morphological changes, HR-HPV clearance rates, pathological remission rates, and side effects after treatment. We also analyzed the possible relationship between HR-HPV subtypes and the efficacy of PDT.

## 2 Materials and methods

### 2.1 Patients

We retrospectively enrolled 96 LSIL patients treated with 5-ALA PDT from January 2019 to June 2022 at the Shanghai Sixth People’s Hospital Affiliated to Shanghai Jiao Tong University School of Medicine. According to 2019 ASCCP guideline, patients diagnosed with low-grade squamous intraepithelial lesions (LSIL) can be managed with clinical observation for the potential for spontaneous remission (2, 13). Patients who have had LSIL persistence for over two years could have treatments with special individual indications, like immunosuppression, cervical surgery history, type 3 transformation zone, and the patients’ urgent desire for invention. In this study, we enrolled patients aged 18–50 years with HR-HPV infection and diagnosed with LSIL (CIN 1) via cervical biopsy, some of which persisted for more than two years. Besides, some of them had a compelling desire for treatment due to anxiety about histological progression as well as preserving cervical integrity and function for conception and fertility expectations. They were also in good compliance and had a favorable economic foundation.

Our primary inclusion criteria were to ensure complete visualization of the cervical squamocolumnar junctions (type 1 transformation zone), and the regions of cervical lesions. We also screened out patients who had undergone previous treatment for CIN, exhibited malignant cells on cytology or histology, displayed suspected cancerous lesions or invasive cancer, or had lesions that had spread to the vaginal epithelium.

The patients were fully informed of the risks and signed the Informed consent before treatment. The ethics committee of Shanghai Sixth People’s Hospital Affiliated to Shanghai Jiao Tong University School of Medicine had approved the study [the IRB number was 2022-KY-031(K)].

### 2.2 5-ALA photodynamic therapy

A sterile cotton, which had been saturated, with a freshly prepared mixture of 20% thermogel (Fudan Zhangjiang Bio-Pharmaceutical Co., Ltd., Shanghai, China) was applied to cover both the cervical surface and canal for 4 hours. The optical source is obtained from a photodynamic therapeutic instrument (LD600c, Wuhan Yage Optic and Electronic Technique Co., Ltd., Wuhan, China). A thin optical fiber was placed in the cervical canal, while a cylindrical head was inserted into the vagina to cover the surface of the cervix. This allowed for light irradiation at 635 nm with a radiant exposure of 100 J/cm2 to be applied to both the cervical surface and canal. Currently, there are no standardized guidelines outlining the recommended concentration and duration of illumination for local cervical application of photosensitizers, nor for the intervals and treatment times for PDT. Therefore, our treatment utilized previous research on cervical diseases and the Chinese expert consensus published in 2022 (12). The treatment was conducted for 30 min per session, with a total of three sessions scheduled once a week. However, it would be postponed for a week during patients’ menstruation. Sexual activity was not allowed during the entire treatment period. Patients were assessed after each session. If any of the following symptoms occurred, the treatment would be stopped: cervical ulcer, continuous cervical bleeding, purulent discharge with a peculiar smell indicating an infection, unrelieved lower abdominal pain, or vulvar pain.

### 2.3 Clinical assessment and outcomes measurement

At 2 weeks checkpoint, we observed the status of the patients’ vulva, vagina, and cervix under colposcopies (whether there was congestion, bleeding, ulcer, infection, etc.), asked the patient whether there were side effects such as abdominal pain and vulvar pain, and treated such symptoms if necessary. At the 3-month checkpoint, all patients underwent a comprehensive evaluation, which included the ThinPrep cytology test (TCT), HPV DNA assay, colposcopy, and histological biopsy. Patients would receive a TCT, HPV DNA Assay, and colposcopy at the 6-month checkpoint. A biopsy would be necessary only if any of these results were positive. In the event that the patient did not attain pathological disappearance during the 3-month checkpoint, a repeat biopsy was performed.

The results of the TCT test (The ThinPrep 2000 system, Hologic Inc., USA) were interpreted using the Bethesda system by pathologists. High-risk HPV infections were detected through HPV DNA Assay (HPV GenoArray Diagnostic Kit, Hybribio Biotech Co., Ltd., Guangdong, China). Cervical biopsy samples underwent evaluation by two pathologists in accordance with the 2014 World Health Organization Classification of female genital tumors (14): (1) normal, (2) low-grade squamous intraepithelial lesion (CIN 1 or others), (3) high-grade squamous intraepithelial lesion (CIN 2 or CIN3), and (4) squamous cell carcinoma.

The effectiveness of PDT was evaluated primarily through biopsy. The histological outcomes were categorized as regression, persistence, and progression. Regression referred to a non-CIN diagnosis, persistence indicated no change in histology, and progression meant an upgrade of the lesion (from CIN 1 to CIN 2, CIN 3, or carcinoma) during follow-up.

### 2.4 Morphological assessment

For each patient, images of the cervix were obtained at the Colposcopy Workstation (x6 magnification level). The iodine-negative areas in the images represent atypical lesions of the cervical epithelium. Image J software (Image J 1.8.0, NIH) was used to calculate the imaging area of the cervix and atypical epithelium. Using a color threshold in our software, we were able to calculate the areas of both atypical and normal epithelium. This allowed us to analyze their irregular shapes in detail and gain a deeper understanding of their characteristics. The iodine-negative area was then divided by the cervical area to obtain the ratio of epithelial atypical lesions.

### 2.5 Statistical analysis

SPSS 22.0 was used for statistical analysis. The chi-square test was used to compare the differences between different clinical data, values of *p* < 0.05 were considered statistically significant.

## 3 Results

### 3.1 Patients’ characteristics

The participants’ ages ranged between 19 and 48 (mean: 28.71 ± 5.21 years old, median: 28 years old). There were 2 people under the age of 20, 58 people aged between 20–29, 33 people aged between 30–39, and 3 people aged between 40–49 (Table 1).

**Table 1:** Baseline characters of participants.

Character	Number
Age	
Median age	28.71 ± 5.21
Age group (years)	
<20	2
20–29	58
30–39	33
40–49	3
≥50	0
Total	96

**Figure 1:**
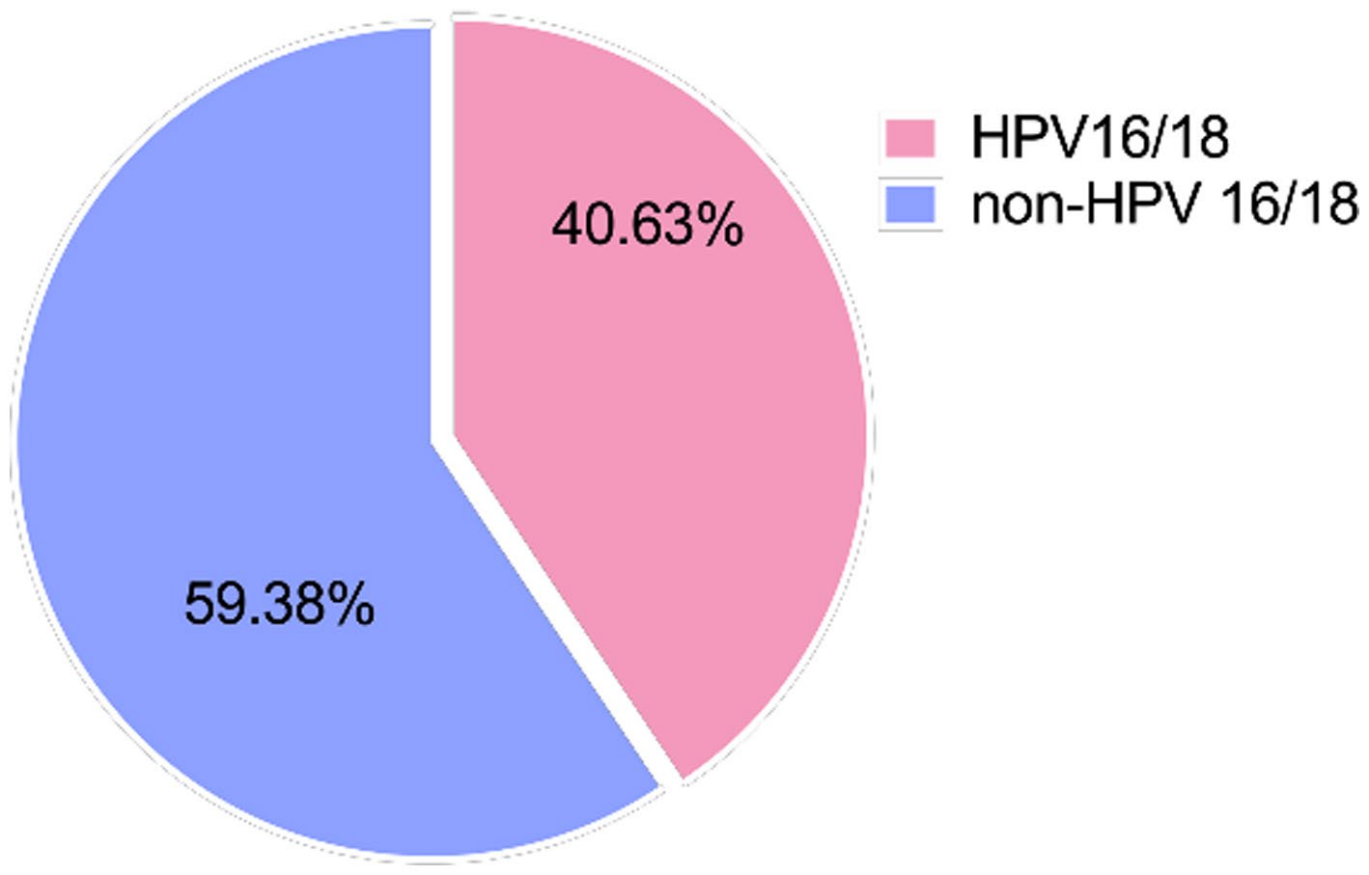
The proportion of HPV 16/18 and non-HPV 16/18 infections.

We further analyzed HPV infection patterns in these 96 patients. In the cohort of 96 patients, 61 (61/96, 63.54%) had a single HR-HPV type, while the remaining 35 (35/96, 36.46%) had two or more types (Table 2). Among the 96 patients, the most prevalent HPV infection genotypes were HPV16 (19.18%) and HPV52 (19.18%), followed by HPV58 (10.27%) and HPV18 (7.53%) (Table 3). In this study, 39 (39/96, 40.63%) patients had HPV infection subtypes associated with HPV 16/18, and 57 cases (59.37%) were related to non-HPV 16/18 subtypes (Figure 1).

**Table 2:** Characters of single and multiple infections in high-risk HPV-infected participants.

Character	Number	Percentage (%)
Infection pattern		
Single infection	61	63.54
Double infection	22	22.92
Triple infection	11	11.46
Multiple infection (≥3)	2	2.08

**Table 3:** Distribution of different HPV genotype infections among recruited patients.

HPV genotype	Number	Percentage (%)
16	28	19.18
52	28	19.18
58	15	10.27
18	11	7.53
51	10	6.85
66	10	6.85
39	9	6.16
56	7	4.79
33	6	4.11
68	6	4.11
82	3	2.05
35	2	1.37
45	2	1.37
53	2	1.37
59	2	1.37
11	1	0.68
31	1	0.68
32	1	0.68
42	1	0.68
81	1	0.68

### 3.2 The colposcopy images of atypical epithelium

**Figure 2:**
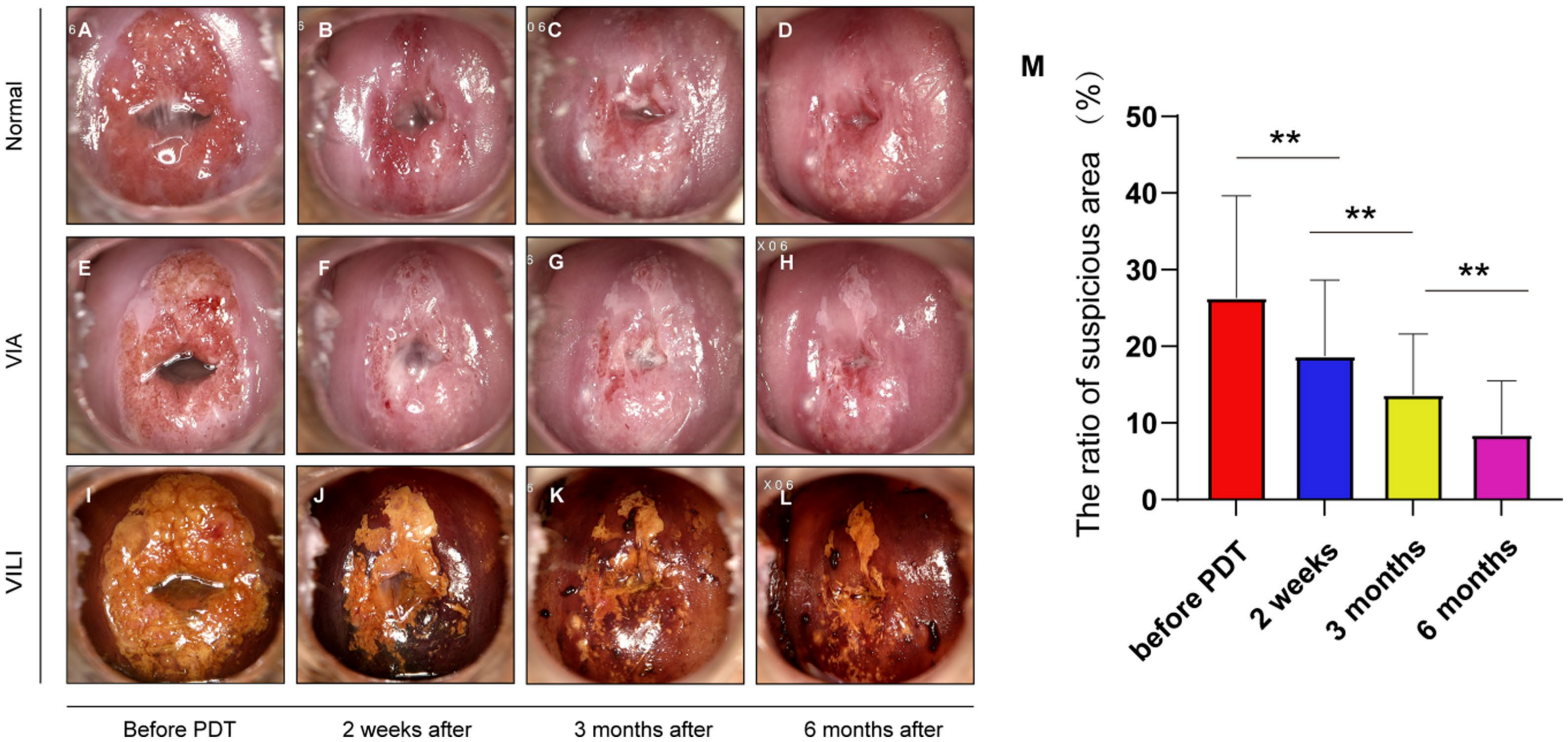
Changes in the area of atypical epithelium under colposcopy. **(A–D)** Pictures of the same patient before treatment, 2 weeks, 3 months, and 6 months after treatment under colposcopy; **(E–H)** pictures of acetowhite test under colposcopy, the time points match the images displayed above; **(I–L)** pictures of Lugol’s iodine test (VILI), the unstained area with iodine decreases over time, matching the time points above; **(M)** the ratios of iodine unstained areas exhibited a significant reduction subsequent to treatment and across the course of time (before treatment, 2 weeks after treatment, 3 months, and 6 months checkpoint) (**p < 0.01).

We conducted colposcopy evaluations before and after 5-ALA PDT to monitor changes in cervical atypical epithelium using VIA (visual inspection with acetic acid) and VILI (visual inspection with Lugol’s iodine). The ratios of atypical epithelial cells indicate the proportion of lesions and regions in the cervix. We found the ratios significantly decreased at 2 weeks, 3 months, and 6 months after PDT (Figure 2).

### 3.3 Histological changes of the cervix

Prior to PDT, 40 out of 96 patients (41.67%) were diagnosed with TCT abnormalities. All the patients have been histologically confirmed with CIN 1. At 3 months checkpoint, all patients were performed with cytological and histological tests. The number of individuals displaying abnormal TCT outcomes dropped from 40 to 11 (11/96, 11.46%)(Table 4). 84 cases (84/96, 87.50%) showed CIN disappearance while 12 cases (12/96, 12.50%) showed CIN persistent (Table 5). At the 6-month checkpoint, 4 patients (4/96, 4.17%) showed abnormal results in the TCT tests (Table 4). All 96 patients underwent colposcopies, with 25 requiring repeated biopsies. Four patients were diagnosed with LSIL (4/96, 4.17%) and one with CIN 2, who underwent LEEP eventually. The total regression rate was 94.79% (91/96) (Table 5).

**Table 4:** The ThinPrep cytology tests of patients before and after ALA-PDT.

TCT	Before ALA-PDT	3 months	6 months
NILM	56	85	92
LSIL	14	8	0
ASC-US	25	3	3
ASC-H	1	0	0
HSIL	0	0	1

**Table 5:** Histological regression at 3 months and 6 months follow-up.

	Total cases	LSIL regression cases	LSIL persistent cases	LSIL progression cases	Regression rate %
3-month checkpoint	96	84	12	0	87.50
6-month checkpoint	96	91	4	1	94.79

In addition, to explore possible factors affecting treatment outcomes, we divided patients into the HPV 16/18-related group (39 patients) and the non-HPV 16/18-related group (57 patients). At the 3-month checkpoint, the pathological regression rate of the HPV 16/18-related group was 89.74% (35/39), and the regression rate of the non-HPV 16/18-related group was 85.96% (49/57) in Table 6. The χ^2^ value was 0.056, *p* = 0.814 > 0.05, no statistical differences were found (Table 6).

**Table 6:** Histological regression at 3 months follow-up (grouped by HPV sub-type).

HPV genotype	Total cases	LSIL regression cases	LSIL persistent cases	LSIL progression cases	Regression rate (%)	χ^2^	*P*
HPV 16/18 related	39	35	4	0	89.74	0.056	0.814
non-HPV16/18 related	57	49	8	0	85.96		

At 6 months checkpoint, the pathological remission rate of the HPV 16/18 related group was 92.31% (36/39), and the remission rate of the non-HPV 16/18 related group was 96.49% (55/57). The χ^2^ value was 0.92, *p* = 0.661 > 0.05, no statistical difference was found (Table 7).

**Table 7:** Histological regression at 6-month follow-up (grouped by HPV 16/18 subtypes).

HPV genotype	Total cases	LSIL regression cases	LSIL persistent cases	LSIL progression cases	Regression rate %	χ^2^	*P*
HPV 16/18 related	39	36	3	0	92.31	0.920	0.661
non-HPV16/18 related	57	55	1	1	96.49		

### 3.4 HPV DNA remission rate

Before 5-ALA PDT treatment, all patients were infected with HR-HPV. The total HR-HPV remission rate was 80.21% (77/96) at 3-month checkpoint and 93.75% (90/96) at 6-month checkpoint. Among them, four patients were observed with new HPV subtypes infection at three months, and one patient was added at six months (Table 8).

**Table 8:** HR-HPV remission rate at 3-month and 6-month checkpoint.

	Total cases	HR-HPV negative cases	HR-HPV persistent cases	HR-HPV genotype changed cases*	Remission rate (%)
3-month checkpoint	96	73	19	4	80.21
6-month checkpoint	96	85	6	5	93.75

^*^It refers to patients who eliminated the previous HR-HPV subtypes after treatment but were infected with new HPV subtypes during follow-ups.

We further explored the effect of HPV sub-types on HR-HPV clearance rate after 5-ALA PDT treatment. At 3 months follow-up, the HR-HPV clearance rate of the HPV 16/18 related group was 94.87% (37/39), the clearance rate of the non-HPV 16/18 related group was 70.17% (40/57), the χ^2^ value was 8.897, *p* = 0.003 < 0.01, according to chi-square test, there was a statistically significant difference between two groups (Table 9).

**Table 9:** HR-HPV remission at 3 months checkpoint (grouped by HPV sub-types).

HPV genotype	Total cases	HR-HPV negative cases	HR-HPV persistent infection cases	HR-HPV genotype changed cases	Remission rate (%)	χ^2^	*P*
HPV 16/18 related	39	33	2	4	94.87	8.897	0.003
non-HPV16/18 related	57	40	17	0	70.17		

At 6 months, the HR-HPV clearance rate of the HPV 16/18 related group was 94.87% (37/39), and that of the non-HPV 16/18 related group was 92.30% (53/57). The χ^2^ value obtained by the Chi-square test was 0, *p* = 1.0 > 0.05, and no significant statistical difference was found (Table 10).

**Table 10:** HR-HPV remission at 6-month checkpoint (grouped by HPV sub-types).

HPV genotype	Total cases	HR-HPV negative cases	HR-HPV persistent infection cases	HPV genotype changed cases	Remission rate %	χ^2^	*P*
HPV 16/18 related	39	33	2	4	94.87	0	1.0
non-HPV 16/18 related	57	52	4	1	92.30		

### 3.5 Safety evaluation

The safety of therapy was evaluated during the entire treatment process. Any significant adverse events would result in an immediate halt to the treatment. No infection or ulcer on the cervix was reported during the 5-ALA PDT treatment. The cervical surfaces appeared smooth and devoid of any scarring after PDT. There were no instances of allergies or severe pain during treatment. Adverse events in our study were as follows: increased discharge (19.79%, 19/96), mild bleeding (8.33%, 8/96), or slight pain (17.71%, 17/96). Most of these adverse events were temporary with spontaneous remission, while some can be relieved after symptomatic treatment in the outpatient department.

## 4 Discussion

The management methods of LSIL patients contain clinical observation, anti-virus drugs, and surgical options. However, the excision or ablation of lesions could affect the cervical integrity and shorten the cervical canal. Non-invasive and tissue-preserving methods including PDT and noninvasive physical plasma (NIPP) treatments etc. are crucial for precancerous lesions of the cervix, as the traditional methods are tissue-destructive, invasive, and associated with side effects such as bleeding, infection, and obstetrical complications. NIPP is a promising transmucosal antineoplastic medical procedure that does not require anesthesia, preserves tissues, and is easy to perform (15). NIPP induces reactive oxygen and nitrogen species (ROS/RNS) that cause DNA damage, resulting in cell cycle arrest and apoptosis (16). NIPP acts through the VIO3/APC3 electrosurgical argon plasma device, which flows through all mucosal layers and induces those cell responses in cells which might cause damage to normal tissues. The PDT works through the accumulation of photosensitizers (PSs), which could be strongly accumulated in pathological tissues. The effect of PDT is closely related to the concentration of photosensitizers and the time of application (17). This mechanism helps PDT specifically targets and inhibits actively proliferating pathological cells, which makes it a promising alternative to cervical precancerous diseases due to its high efficacy and minimal side effects (18). Following the administration of exogenous PSs, mitochondria experience phototoxicity in pathological cells but less in normal cells (19). These non-invasive treatments barely induce ulcers or scar formation, which is beneficial to preserving fertility and preventing the occurrence of cervical insufficiency.

Our study found that the cervix could maintain its integrity with anatomical structure while the area of the cervical atypical epithelium was significantly reduced after 5-ALA PDT treatment. Meanwhile, Mazdziarz et al. (20) found that 10 patients treated with 5-ALA PDT due to CIN or VIN had successful pregnancies and gave birth to healthy full-term infants, suggesting that PDT has no adverse effects on pregnancy. Recent studies suggested that 5-ALA PDT can achieve a promising histological remission rate for LSIL. Li et al. (11) found that the complete remission rates of CIN 1 were 88.31 and 94.81% at 6 months and 12 months after 5-ALA PDT in 77 patients. Gu et al. (9) reported that 258 patients with LSIL combined with HR-HPV infection had a pathological remission rate of 84.88% in 6 months.

Compared with traditional methods, PDT can reach a similar pathological remission rate. The effect of 5-ALA PDT was similar to CO2 laser in four to six months (84.7 and 83.1%), but the long-term efficacy of 5-ALA PDT was better than CO2 laser (87 and 67.3%) (21). CO2 laser therapy may cause pain, tissue scarring and residual lesions, affecting sexual quality of life (22). Fallani et al. found that one out of five CO2 laser treated patients suffered from HPV persistence (23). The standard medical procedure for treating cervical precancerous lesions is cervical conization, including LEEP (loop electrosurgical excision procedure) and CKC (cold-knife conization). Bodner et al. (24) indicated that 100% vs. 91% (CKC vs. PDT) of the patients were disease-free, and no statistically significant differences concerning HPV eradication and recurrence between the two methods. Cervical conization can have a negative impact on the integrity of the cervix, which may result in obstetric complications like cervical insufficiency. If recurrence occurs, additional surgery such as another conization or a hysterectomy may be necessary. This could lead to psychological distress for the patients and their family. In consistent with the above studies, we found that the pathological remission rate reached 87.5% (84/96) at the third month and 94.79% (91/96) at the 6th month after treatment in LSIL patients. Furthermore, we also found that the pathological remission rate reached 81.82% at the third month and 90.91% at sixth month in HSIL patients (25). Herein, we believe that this non-surgical and repeatable intervention is more effective in the treatment of LSIL than traditional therapies. Given that the missing diagnoses of HSIL account for a certain proportion of LSIL patients, PDT can prevent the progression of LSIL to HSIL or even cervical cancer by simultaneously acting on clinical overt lesions, subclinical lesions, and latent infection.

5-ALA PDT also has a good clearance rate against HR-HPV infection. Studies showed that 5-ALA, the metabolic precursor of photosensitizer PpIX (26), can highly be accumulated in HPV-infected cells, and PDT can specifically inhibit the proliferation ability of HPV-positive cervical cells without destroying normal cells (27). We observed the overall HR-HPV clearance rate of cervical LSIL was 80.21% (77/96) 3 months after 5-ALA PDT treatment, and 93.75% (90/96) at 6 months follow-up.

However, it has not been concluded whether the efficacy of 5-ALA PDT in the treatment of cervical LSIL is related to HPV infection subtypes. It is known that HPV 16 and 18 are the most pathogenic subtypes, and about 71% of cervical cancers are associated with these two subtypes (28). Whether a patient is infected with HPV 16/18 directly affects the regression rate of CIN lesions in patients. Patients with HPV 16/18 infection are more likely to benefit from PDT treatment. Cang et al. (29) reported that the HPV clearance rate in the HPV 16/18-related group was 75.0%, and that of the other 12 high-risk types of infection group was 48.8% three months after PDT. However, a retrospective study found that HPV clearance at 3 months after PDT in 258 patients with cervical LSIL was not correlated with HPV16/18 infection (9). Interestingly, we found different HPV subtypes might have an impact on the therapeutic effect of PDT in the short term, but for the long-term effect, the virus clearance rate of patients with different HPV subtypes gradually tends to be the same. The clearance rate of the HPV 16/18 group [94.87% (37/39)] was significantly higher than that of the non-HPV 16/18 group [70.17% (40/57)]. However, at 6 months after treatment, the clearance rate of the HPV 16/18 group was still 94.87% (37/39), while that of the non-HPV 16/18 group increased to 92.30% (53/57), showing no statistical difference. Studies have found that PDT has anti-inflammatory and antimicrobial properties (30). Researchers also indicated that the immune system could clear penetration and integration of HPV, and persistent HPV infection is associated with the ability to escape immune clearance (31). Under specific wavelengths of light sources, PDT produces cytotoxic substances targeting photosensitizers-accumulated pathological cells, induces microvascular damage, and stimulates immune responses (32). In that way, we proposed a hypothesis that this HPV subtype’s independent therapeutic effect is related to the establishment of long-term immune effects after treatment. In our previous study, we found a significant difference in the expression of CD4+ and CD8+ T cells before ALA-PDT and at 3 months follow-up, which indicated that PDT could boost specific cellular immunity to eliminate HPV infection (25).

In addition, the effect of PDT is also closely related to the concentration of photosensitizers, which are strongly accumulated in pathological tissues, and the duration of illumination. The effect of PDT is closely related to the concentration of 5-ALA and the time of application, which determined whether enough photosensitizer PpIX is accumulated in cervical epithelium (17). The treatment conditions and frequency of PDT treatment for cervical diseases in our outpatient department are all selected by referring to the Chinese expert consensus in female lower genital tract diseases (2022) and previous published research articles on cervical diseases (12). A group-controlled study found that the application of 5-ALA at a concentration of 10–20% was safe for patients, and the cervical tissue absorption of 5-ALA at a concentration of 20% reached a peak 6 h after local administration, and the intratissue accumulation and transformation of 5-ALA could not be increased even after prolonged application time or increased concentration (17). In this study, We applied a thermogel mix containing 20% 5-ALA to cover both the cervical surface and canal for a duration of 4 h. During photodynamic therapy, only a minority of patients experienced mild adverse reactions, including increased vaginal secretions, slight pain or lower abdominal swelling, and a small amount of vaginal spotting bleeding. These adverse reactions could be relieved spontaneously and need no additional interventions. No patient discontinued treatment due to adverse complications. Nonetheless, recent studies have found that ALA can produce protoporphyrin which accumulates in normal cells, potentially causing cytotoxicity to the surrounding healthy cells (33). The cellular biology mechanisms need further exploration.

One disadvantage of this research is that it has a relatively small sample size. This is mainly because Photodynamic therapy (PDT) is a costly and time-consuming treatment option that demands strict adherence and financial support. The PDT treatment procedure requires weekly sessions that may cause transportation inconvenience and scheduling conflicts. The study is also limited by its single-center non-randomized design and inadequate follow-ups.

In that way, we will further expand the number of cases, set up a control group, and conduct longer follow-ups, including the recurrence and the impact of 5-ALA PDT on the fertility and delivery outcomes of patients, to provide more evidence for 5-ALA PDT in the treatment of cervical diseases.

## 5 Conclusion

5-ALA PDT has a good therapeutic effect and safety in the treatment of LSIL patients with HR-HPV infection. In the future, a multicenter, prospective, randomized controlled trial with long-term follow-up should be conducted to further explore the potential applications of other female lower genital tract diseases including cervical high squamous intraepithelial lesions (HSIL), vaginal SIL, vulvar SIL, vulvar lichen sclerosus (VLS), and condyloma acuminatumon (CA).

## Data availability statement

The raw data supporting the conclusions of this article will be made available by the authors, without undue reservation.

## Ethics statement

The ethics committee of Shanghai Sixth People’s Hospital Affiliated to Shanghai Jiao Tong University School of Medicine had approved the study [the IRB number was 2022-KY-031(K)]. The studies were conducted in accordance with the local legislation and institutional requirements. The human samples used in this study were acquired from primarily isolated as part of your previous study for which ethical approval was obtained. Written informed consent for participation was not required from the participants or the participants’ legal guardians/next of kin in accordance with the national legislation and institutional requirements.

## Author contributions

YL: Writing – original draft. JC: Writing – original draft. YH: Writing – original draft. QX: Writing – original draft. RJ: Writing – original draft. YT: Project administration, Writing – review & editing. YX: Conceptualization, Writing – review & editing. LM: Project administration, Writing – review & editing.
